# Structural organization and interactions of transmembrane domains in tetraspanin proteins

**DOI:** 10.1186/1472-6807-5-11

**Published:** 2005-06-28

**Authors:** Oleg V Kovalenko, Douglas G Metcalf, William F DeGrado, Martin E Hemler

**Affiliations:** 1 Department of Cancer Immunology and AIDS, Dana-Farber Cancer Institute and Department of Pathology, Harvard Medical School, Boston, USA; 2 Department of Biochemistry and Biophysics, School of Medicine, University of Pennsylvania, Philadelphia, USA; 3 Dana-Farber Cancer Institute, D-1430, 44 Binney Street, Boston, MA 02115, USA

## Abstract

**Background:**

Proteins of the tetraspanin family contain four transmembrane domains (TM1-4) linked by two extracellular loops and a short intracellular loop, and have short intracellular N- and C-termini. While structure and function analysis of the larger extracellular loop has been performed, the organization and role of transmembrane domains have not been systematically assessed.

**Results:**

Among 28 human tetraspanin proteins, the TM1-3 sequences display a distinct heptad repeat motif (***abcdefg***)_n_. In TM1, position ***a ***is occupied by structurally conserved bulky residues and position ***d ***contains highly conserved Asn and Gly residues. In TM2, position ***a ***is occupied by conserved small residues (Gly/Ala/Thr), and position ***d ***has a conserved Gly and two bulky aliphatic residues. In TM3, three ***a ***positions of the heptad repeat are filled by two leucines and a glutamate/glutamine residue, and two ***d ***positions are occupied by either Phe/Tyr or Val/Ile/Leu residues. No heptad motif is apparent in TM4 sequences. Mutations of conserved glycines in human CD9 (Gly25 and Gly32 in TM1; Gly67 and Gly74 in TM2) caused aggregation of mutant proteins inside the cell. Modeling of the TM1-TM2 interface in CD9, using a novel algorithm, predicts tight packing of conserved bulky residues against conserved Gly residues along the two helices. The homodimeric interface of CD9 was mapped, by disulfide cross-linking of single-cysteine mutants, to the vicinity of residues Leu14 and Phe17 in TM1 (positions ***g ***and ***c***) and Gly77, Gly80 and Ala81 in TM2 (positions ***d***, ***g ***and ***a***, respectively). Mutations of ***a ***and ***d ***residues in both TM1 and TM2 (Gly25, Gly32, Gly67 and Gly74), involved in intramolecular TM1-TM2 interaction, also strongly diminished intermolecular interaction, as assessed by cross-linking of Cys80.

**Conclusion:**

Our results suggest that tetraspanin intra- and intermolecular interactions are mediated by conserved residues in adjacent, but distinct regions of TM1 and TM2. A key structural element that defines TM1-TM2 interaction in tetraspanins is the specific packing of bulky residues against small residues.

## Background

Tetraspanins constitute a large family of integral membrane proteins, characteristically containing 4, 6 or 8 conserved cysteine residues in the large extracellular loop (including the CCG and PxxCC motifs), which form disulfide bonds, and several conserved polar residues in the intracellular loop and transmembrane regions [1,2]. There are 32 putative tetraspanin family members in mammals, 37 in *Drosophila melanogaster *and 20 in *Caenorhabditis elegans*. Tetraspanins play diverse roles in cell adhesion, migration and fusion processes, cellular activation and signaling (reviewed in refs. [2-4]). Mammalian tetraspanins such as CD9, CD63, CD81, CD82, CD151, rds/peripherin, and uroplakins Ia and Ib have been most extensively studied, with mouse knock-out models available for CD9 [5-7], CD81 [8,9], CD151 [10] and a few others. However, the majority of tetraspanins are characterized very little, if at all, at genetic, biochemical or structural levels.

The large extracellular loop (LEL) of tetraspanins has received most attention, since it contains functionally important sites. Sequence QRD (194–196) in CD151 is important for association with integrins, which has functional consequences for integrin-dependent cell spreading and multicellular cable formation [11]. A site in the LEL of CD9, SFQ (residues 173–175), is essential for CD9 function in sperm-egg fusion [12]. The crystal structure of tetraspanin CD81 LEL revealed five α-helixes, A-E [13]. Helices A, B and E form a relatively conserved region in tetraspanins, whereas the region between helices B and E is the most variable [14]. Interestingly, the variable region contains most of the functionally important sites involved in tetraspanin protein-protein interactions.

A remarkable biochemical property of tetraspanin molecules is their ability to associate with a large number of other transmembrane proteins, including integrins, membrane-associated growth factors and receptors, MHC class II molecules, Ig superfamily proteins, and each other [2,3,15]. Several of these lateral associations of tetraspanins are detected in "mild" detergents (Brij series, CHAPS), but are disrupted by "strong" detergents such as Triton X-100 or SDS. Multiprotein complexes of tetraspanins and associated molecules, also called the "tetraspanin web" [16], may represent a distinct tetraspanin-enriched membrane microdomain [17,18]. The formation of this microdomain is influenced by palmitoylation of several conserved juxtamembrane cysteine residues in tetraspanins [19-21].

The transmembrane domains, encompassing nearly half of a tetraspanin protein, are the most conserved part of the molecule (Stipp *et al. *[1] and this study). However, very little functional information is available on these domains. The differential detergent sensitivity of tetraspanin-tetraspanin associations suggests that hydrophobic interactions between TM helices may play a role. Indeed, when the large extracellular loop (LEL) of CD151 is deleted, the molecule is still able to associate with other tetraspanins [22]. Thus, TM domains are strong candidates for mediating tetraspanin-tetraspanin interactions.

The importance of TM domain interactions in intramolecular organization was demonstrated in a study showing that CD82 fragment TM2-4, lacking TM1, was retained in the endoplasmic reticulum, but was transported to the cell surface upon co-expression of TM1 [23]. This *in vivo *reconstitution experiment demonstrated a strong interaction between TM1 and the rest of the molecule. Expression of a truncated CD9 molecule (TM3-LEL-TM4) results in intracellular accumulation of the protein and significant misfolding of the LEL, as judged by inappropriate disulfide formation and diminished antibody reactivity (our unpublished data). Similarly, a CD9 epitope in the LEL is lost in molecules lacking either TM2+TM3 or just TM4 [24]. Thus, TM domain interactions and packing are crucial for proper folding, stability and transport of tetraspanin molecules.

In a previous study, we showed that covalent cross-linking of membrane-proximal cysteine residues can be used as a tool for detection of tetraspanin-tetraspanin associations [25]. Inhibition of cysteine palmitoylation by 2-bromopalmitate (2-BP) made cysteines available for cross-linking and enabled demonstration of specific tetraspanin homodimerization and low levels of heterodimerization. We concluded that tetraspanin homodimers, formed in the Golgi, may be a fundamental structural unit within tetraspanin microdomains.

In this study, we carried out detailed sequence analysis of human tetraspanin TM domains. We show that a heptad repeat containing conserved glycine, asparagine and large hydrophobic residues occurs in TM1 and TM2 domains, and predict tight intramolecular association of these two domains by packing of the large residues against the small residues. Moreover, by using cysteine cross-linking we map a dimerization interface in the human CD9 protein, and show that conserved heptad motif glycine residues are also important for intermolecular CD9 associations.

## Results

### Sequence analysis of tetraspanin transmembrane domains: presence of the heptad repeat motif

We focused our attention on 28 human tetraspanins identified from the SWISS-PROT and GenBank databases. All tetraspanins have in common four hydrophobic stretches (TM domains) of 20–25 residues, and contain highly conserved residues in the second extracellular loop, in particular the Cys-Cys-Gly (CCG) motif. Detailed analysis of the large extracellular loop sequences [14], and dendrograms based on full-length alignment can be found in earlier studies [26,27]. The length of each transmembrane domain was established based on previous sequence analysis of tetraspanin sequences [27,28], and on annotations to the database entries. Manual adjustments based on sequence homology and hydrophobicity profiles were done to fully delineate the TM domains. The resulting lengths of TM domains were: TM1 – 23 residues; TM2 – 21 residues; TM3 – 25 residues; TM4 – 25 residues. Two more residues could be added onto the N-terminal part of TM2; however, relatively small sequence conservation of these residues among tetraspanins and occurrence of polar/charged side chains in some tetraspanins precluded us from doing so for the global alignment.

**Figure 1:**
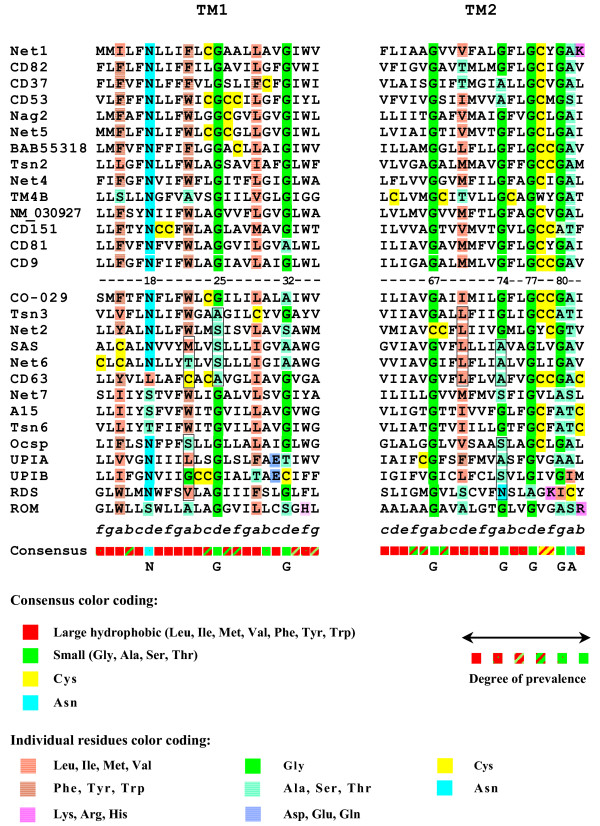
**Sequence alignment of the transmembrane domains 1 and 2 of 28 human tetraspanins**. Residues from select positions of the heptad motifs in TM1 and 2 are highlighted (see text for details). Also highlighted are polar residues and cysteines. Consensus residue types are shown by the color scheme indicated. Boxed residues reflect correlated substitutions for position pairs 22–74 and 25–70 (details are in the text). The numbers refer to CD9 sequence.

**Figure 2:**
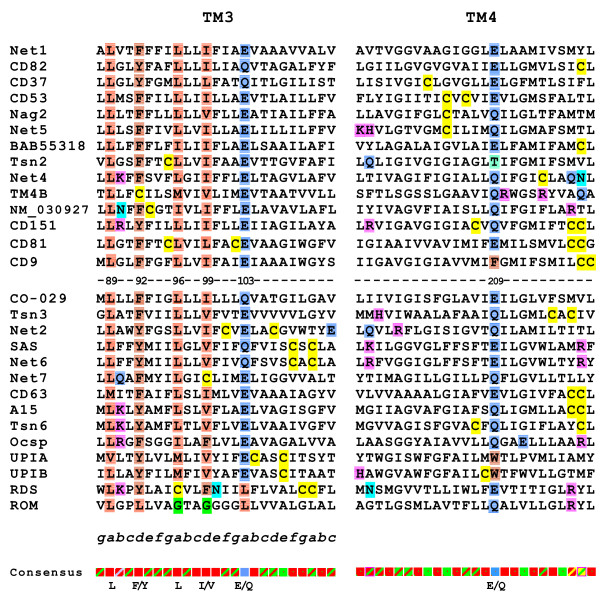
**Sequence alignment of the transmembrane domains 3 and 4 of 28 human tetraspanins**. Residues from heptad positions ***a ***and ***d ***in TM3 are highlighted. Also highlighted are the conserved Glu/Gln residue in TM4, other polar residues and cysteines. The color scheme is as in Figure 1.

Figures 1 and 2 show a multiple sequence alignment of four TM domains of 28 human tetraspanins. For each position within the domains, consensus residues were determined and classified (with individual color code) in 4 categories: 1) large hydrophobic residues (including Val, Met, Leu, Ile, Phe, Tyr, Trp), 2) small residues (Gly, Ala, Ser and Thr), 3) Cys, and 4) Asn. When more than two types of residues occupied a given position in a TM, a dual-color pattern that reflected the prevalence of the particular residue type was used (Figure 1). Cysteine residues were shown separately due to their importance as palmitoylation target sites. The highly conserved asparagine residue in TM1 was considered separately. No proline residues are found in TM domains 1–3 of human tetraspanins.

An inspection of the multiple sequence alignment reveals a repeating heptad amino acid pattern, (***abcdefg***)_n_, in TM1, 2 and 3 (Figure 1, 2). Heptad repeats promote helical coiled coil interactions in multiple soluble and membrane-spanning proteins [29-31]. In the heptad repeat, hydrophobic residues in positions ***a ***and ***d ***are of special importance, as they directly mediate interhelical contacts by creating a tight knobs-into-holes packing in the coiled coil structure [32]. For instance, in the leucine zipper of the yeast transcription factor GCN4, positions ***a ***and ***d ***contain Val and Leu residues, respectively, with an Asn residue in a single ***a ***position forming a hydrogen bond across the GCN4 dimer interface [33].

**Figure 3:**
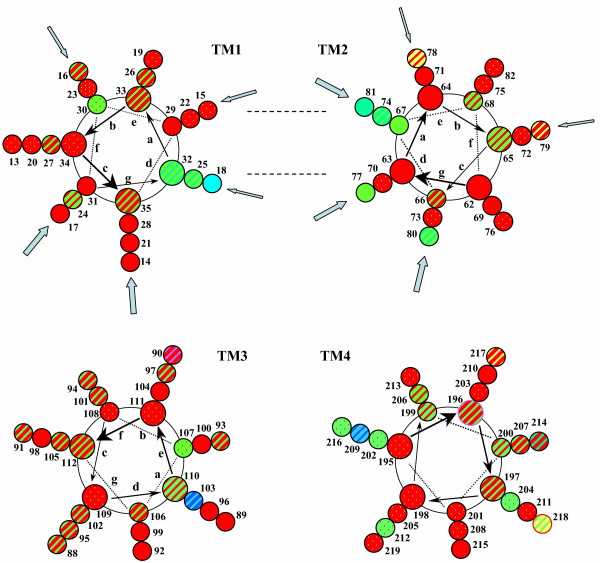
**Helical wheel diagrams of transmembrane domains TM1-4 reflecting the consensus residue types**. The color scheme is as in Figure 1. The numbers refer to CD9 sequence. Heptad positions ***a ***through ***g ***are indicated for TM1-3. A predicted interaction between positions ***a ***and ***e ***in TM1 and ***a ***and ***d ***in TM2 is shown by dotted lines (see Figure 6 and text for details). Arrows reflect the efficiency of intermolecular cross-linking via single cysteines placed in these positions (see Figure 7 and text for details).

In TM1 of tetraspanins, highly conserved Asn, Gly and Gly residues (numbers 18, 25 and 32 in the CD9 sequence) appear at ***d ***positions of the heptad repeats, and residues 14, 21 and 28 are at ***a ***positions (Figure 1). In TM2, residues 67, 74 and 81 (consensus Gly, Gly and Ala, respectively) occupy ***a ***positions, whereas residues 63, 70 and 77 are at ***d ***positions. Another highly conserved glycine, Gly80, occupies the 3^rd ^***g ***position in TM2. In TM3, the conserved pattern consists of two leucine residues (Leu89 and Leu96) and a glutamate/glutamine residue (Glu/Gln103) in ***a ***positions (Figure 2). Two ***d ***positions are also conserved – Phe/Tyr92 and Ile/Val/Leu99. TM4 lacks a conserved heptad pattern and has only a single conserved position, Glu/Gln209 (with four exceptions). These features of TM1-4 of tetraspanins are displayed on helical wheel diagrams (Figure 3).

### Analysis of TM1 sequences

**Figure 4:**
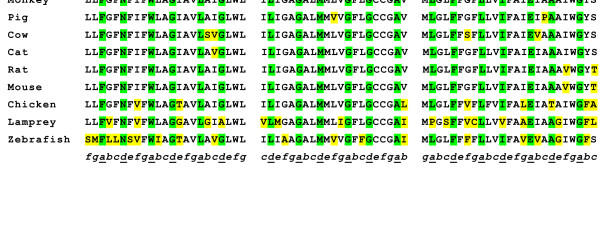
**Sequence alignment of TM1-3 for ten vertebrate orthologs of CD9**. Heptad positions ***a ***and ***d ***in TM1, TM2 and TM3 are highlighted in green. Residues that differ between orthologs are shown in yellow.

The conserved Asn-Gly-Gly motif, occupying designated ***d ***positions of the heptad repeat, is the most prominent structural feature of TM1. We also compared sequences of CD9 orthologs from 10 different organisms (the most available for any tetraspanin) to gain further insight into conservation and variability of the TM1 sequence. As shown in Figure 4, positions ***a***, ***d ***and ***g ***in TM1 are among the most conserved (0, 1 and 1 substitution, respectively), while interspecies variability tends to occur in other positions: ***b ***(5 substitutions), ***c ***(4 substitutions), ***e ***(4 substitutions) and ***f ***(4 substitutions). Thus, the positions typically involved in coiled coil interactions (***a ***and ***d***) are the most conserved.

When residues of TM1 are plotted as a helical wheel, additional structural features are revealed (Figure 3). There are highly conserved aliphatic and aromatic residues in the first three ***a ***positions of the heptad motif (Phe15, Trp22 and Leu29 in CD9), as well as in ***g ***positions (Leu14, Phe21, Val28 in CD9). The "ridges" formed by these bulky residues are flanking the "groove"-forming Gly residues of the Asn-Gly-Gly position ***d ***motif. In contrast, ***b***, ***c***, ***e ***and ***f ***positions show an overall higher variability among tetraspanins, as also seen in the comparison of CD9 orthologs described above.

### Analysis of TM2 sequences

A landmark feature of TM2 in tetraspanins is the presence of highly conserved glycine residues (Gly67, 74, 77 and 80 in CD9, Figure 1). Other substitutions at these positions are almost exclusively small residues, such as Ala or Ser. In addition, Ala, Ser or Thr occupy position 81. This residue, together with Gly67 and Gly74, forms face ***a ***of the helix. Residue Gly77 (position ***d***) is preceded by conserved, chiefly large hydrophobic residues on the same helical face (Leu63 and Met70 in CD9). Extremely conserved Gly80 falls into heptad position ***g ***(Figure 3). Among CD9 orthologs, heptad positions ***a ***and ***d ***are absolutely conserved, whereas other positions have the following number of substitutions: ***b ***– 3; ***c ***– 2; ***e ***– 1; ***f ***– 3; ***g ***– 1 (Figure 4). Two of the ***f ***position residues in TM2 (65 and 79) also show higher variability among different tetraspanins (Figures 1, 3). Cysteine residues are frequently found near the cytoplasmic end of TM2 helix at positions 78 and 79; these cysteines are likely to be palmitoylated.

### Analysis of TM3 and TM4 sequences

The TM3 domain provides another example of the heptad repeat pattern. Position ***a ***is occupied by two highly conserved leucine and a glutamate/glutamine residue (Leu89, Leu96 and Glu/Gln103 in CD9). Furthermore, two ***d ***positions are conserved – Phe/Tyr92 (aromatic residue) and Ile/Val99 (β-branched aliphatic residue; Figures 2, 3). In addition, residue 100 in position ***e ***is generally Phe or Leu. Among CD9 orthologs, position ***a ***has 1 substitution, positions ***b***, ***c ***and ***f ***each have 6, positions ***d ***and ***e ***each have 2, and ***g ***has 4. Thus, as for TM1 and TM2, positions ***a ***and ***d ***are among the most conserved, but overall TM3 has more variable positions than TM1 or TM2 (Figure 3). Less than half of TM3 sequences contain cysteine residues, and those tend to occur at the internal positions of the helix (Figure 2).

TM4 shows less conservation among various tetraspanin family members than the other TM domains (Figures 2, 3). The only highly conserved feature is the glutamate/glutamine residue in position 209. In addition, one or two cysteine residues can be found at the C-terminal end of TM4 in some tetraspanins (e.g. CD9, CD81, CD151), and many sequences contain additional polar residues (Arg, Lys, His, Asn, Gln). No conserved heptad motif was identified in TM4, as also confirmed by analysis of substitutions in CD9 orthologs (data not shown).

### Mutational analysis of conserved glycine residues in TM1 and TM2

**Figure 5:**
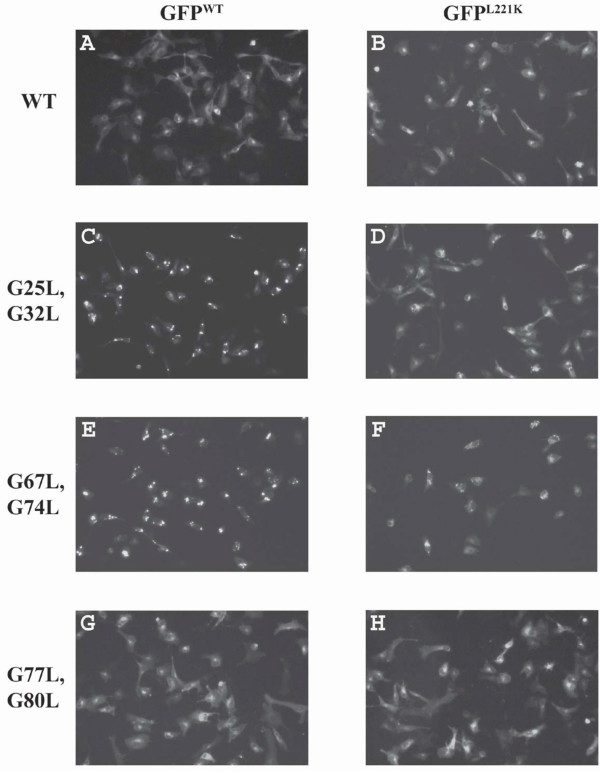
**Expression of wild-type and mutant CD9 TM(1+2)-GFP proteins in human cells**. Human rhabdomyosarcoma RD cells were transfected with constructs encoding CD9 TM(1+2)-GFP fusion proteins that carried mutations indicated. Either wild-type or monomeric (L221K) GFP was used. Images were captures 18–28 hours post-transfection.

The conserved nature of the Asn and Gly residues in TM1 and TM2 prompted an analysis of their functional role. To this end, we have probed whether mutations of these residues destabilize the protein molecule. We expressed a construct of the first and second TMs of CD9, connected by the small extracellular loop, and tagged with a C-terminal green fluorescent protein (TM(1+2)-GFP molecule). In human rhabdomyosarcoma RD cells, the wild-type fusion protein localized mostly in a reticular, intracellular pattern, without forming any large aggregates (Figure 5, panel A). Remarkably, when double mutants Gly25Leu + Gly32Leu and Gly67Leu + Gly74Leu were expressed, the protein formed distinct large aggregates in a high proportion of cells (Figure 5, panels C and E). In contrast, double mutant Gly77Leu + Gly80Leu did not form such aggregates (Figure 5, panel G). Results with respective single mutants were similar to that with double mutants, with the aggregation being somewhat more pronounced for Leu substitutions of Gly67 and Gly74 compared to Gly25 and Gly32 mutations. No aggregation was observed for Asn18Ser and Asn18Tyr mutants (data not shown). Also, nearly identical results were obtained with human HT1080 cells (data not shown).

We interpret these results as an indication that aggregating mutants are destabilized or misfolded while non-aggregating mutants retain the wild-type conformation. Intriguingly, mutations to the conserved GG7 motifs caused protein aggregation while the mutation of other glycines had no detectable effect. These results also suggest that wild-type GFP, which has weak tendency to self-associate, could enhance non-specific interactions of destabilized mutant TM(1+2) CD9 moieties, leading to their aggregation. Consistent with this hypothesis, the aggregation of Gly25Leu + Gly32Leu and Gly67Leu + Gly74Leu double mutants was suppressed when monomeric GFP molecule, Leu221Lys [34] was used (Figure 5, panels D and F). The use of monomeric GFP did not affect intercellular localization of wild-type CD9 TM(1+2) (Figure 5, panel B), or a Gly77Leu + Gly80Leu double mutant (Figure 5, panel H).

In summary, Leu substitutions of Gly residues that are part of the Asn-Gly-Gly (NGG7) motif in TM1, or Gly-Gly-Ala (GGA7) motif in TM2, resulted in destabilization and aggregation of GFP-fused TM(1+2) proteins, whereas substitutions of Gly77 or Gly80, which are not part of these motifs (Figure 3), failed to show such aggregation.

### Prediction and modelling of interaction between TM1 and TM2

Consecutive helices in polytopic membrane proteins frequently interact [35]. Sequence analysis of TM1 and TM2 helices of tetraspanins reveals a remarkable complementarity in the distribution of large and small residues at heptad positions ***a ***and ***d ***along the helical axis (Figure 3), suggesting that these residues may interact. To further elucidate the potential for TM1-TM2 interaction, the putative interface was modeled using a novel algorithm that considers mutational data during each step of a Monte Carlo simulated annealing cycle (see *Methods *for details). Specifically, Gly25Leu, Gly32Leu, Gly67Leu and Gly74Leu were scored as disruptive mutations, while Asn18Ser, Gly77Leu and Gly80Leu were scored as silent mutations, based on their effects on protein stability (Figure 5 and data not shown).

**Figure 6:**
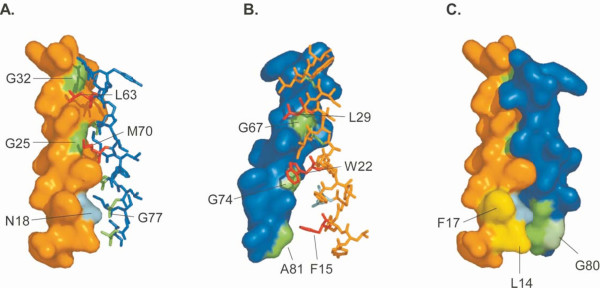
**Structural model of TM1-TM2 interaction in CD9**. Shown are space-filling models of CD9 TM1 (panel A), TM2 (panel B), and the two helices together (panel C). Small and large residues of heptad positions ***a ***and ***d***, which form the crucial contacts between the helices, are shown in green and red, respectively. Asn18 is shown in blue, Leu14 and Phe17 in yellow, and Gly80 in light green.

The resulting model predicts left-handed crossing of TM1 and TM2 helices at an angle of +28°. The key element of the structure is the apposition of bulky and small heptad position ***a ***and ***d ***residues, as follows: Gly32-Leu63; Gly67-Leu29; Gly25-Met70; Gly74-Trp22; Asn18-Gly77; Ala81-Phe15 (Figure 6). Our model predicts that each of these residue pairs are in van der Waals contact. Additionally, two potential H-bonds are predicted in this model, indicating close packing: Gly67 C_α _to Gly25 carbonyl oxygen, and Trp22 C_α _to Met70 carbonyl oxygen. The packing is tighter in the ectodomain-proximal portion of the helices (Figure 6, panel B), as determined by C_α_-C_α _distances between interacting residue pairs.

The key elements of the model are corroborated by the presence of apparently complementary substitutions in TM1 and TM2 sequences of different tetraspanins (Figure 1, boxed residues). For example, Gly74 is predicted to interact with Trp22. In 8 of the 10 tetraspanins that contain a substitution for Gly74, a compensatory substitution occurs at the Trp22 position (Figure 1). Thus, a larger non-glycine side chain at position 74 may necessitate a less bulky non-Trp side chain in position 22. Likewise, the presence of a C_β _at position 25, typically occupied by glycine, necessitates a non-β-branched amino acid at position 70, which is occupied by a β-branched residue in nearly half of all cases. Indeed, we find that in each of 5 cases in which position 25 contains a C_β_, a leucine residue occurs in position 70. This analysis is consistent with our molecular model that suggests Leu70 will pack most favorably against a C_β _at position 25 than a β-branched residue or a methionine.

### Role of TM1 and TM2 heptad motif residues in CD9 dimerization

To probe CD9 dimerization, we used a cysteine-mediated cross-linking approach. We established previously a simple and efficient method for cysteine-mediated cross-linking [25]. After cells are pre-treated with 2-BP for 16–24 hours to expose normally palmitoylated cysteines, the cysteines can be cross-linked using any of the following methods: a) Spontaneous oxidation in Brij97 lysates (a condition that preserves tetraspanin-tetraspanin associations), b) *In situ *cross-linking, by pre-lysis oxidation of cells with Cu^2+^-phenanthroline (CuP) to promote disulfide bond formation. c) *In situ *cross-linking with thiol-reactive cross-linking agents of defined length (e.g. DTME, BMB). The first two approaches produce in essence "zero-length" disulfides, indicative of close proximity of target cysteines and presumably high specificity of interaction. In contrast, chemical cross-linkers with 6–20 Å spacer arm may cross-link with higher efficiency, but not necessarily higher specificity. However, they provide advantages such as variable membrane permeability, and potential linkage cleavability. For tetraspanins such as CD9, membrane-permeable cross-linker DTME (13.3 Å-long, reducible) provides highly specific and efficient cross-linking [25]. Here we have used a cysteine cross-linking strategy, in combination with cysteine-scanning mutagenesis, to map the residues from TM1 and TM2 contributing to the CD9 dimerization interface.

For subsequent cross-linking experiments using CD9 TM(1+2)-GFP protein, the non-dimerizing form of GFP was used. This avoids potential GFP-dependent dimerization and aggregation that can be observed with wild-type GFP, especially when fusions with transmembrane proteins are studied [36]. Importantly, the Leu221Lys mutation in GFP prevented aggregation of mutant forms of CD9 TM(1+2), which was observed with wild-type GFP fusion (Figure 5). The TM(1+2) fragment of CD9 contains three native cysteines – Cys9, Cys78 and Cys79. Single-cysteine mutants of TM(1+2) were constructed, in which a cysteine was placed at various faces of TM1 or TM2 while all of the wild-type cysteines were simultaneously replaced by serines. The mutant proteins were transiently expressed in RD cells (having little endogenous CD9), which were then treated for 16–18 hours with 2-BP. To achieve maximal specificity in cross-linking we used a "zero-length" agent, CuP.

**Figure 7:**
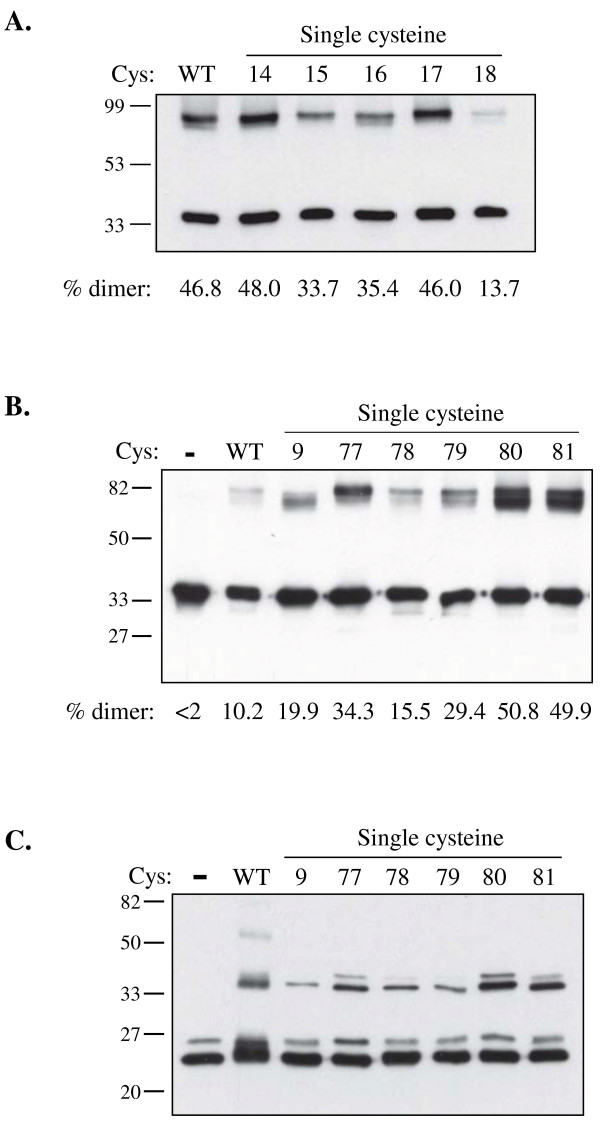
**Cross-linking of single-cysteine mutants of CD9 protein**. GFP fusions of CD9-TM(1+2) protein (panels **A **and **B**) or untagged full-length CD9 (panel **C**), and containing wild-type cysteines (Cys9,78,79), a single cysteine at positions indicated, or no cysteines (all replaced by Ser) were transiently expressed in human RD cells and cross-linked using CuP. Cell lysates were analyzed by Western blotting for GFP (panels **A **and **B**) or CD9 (panel **C**). % dimer is the fraction of dimer in total protein material (monomer + dimer), based on densitometry measurements of respective bands.

First, single-cysteine replacements were constructed for residues Leu14, Phe15, Gly16, Phe17 and Asn18, covering just over one complete helical turn at the beginning of TM1. While residue Asn18 is highly conserved, positions 14, 15 and 17 are occupied by bulky hydrophobic residues in most tetraspanins, whereas position 16 shows less conservation (Figures 1, 4). All of the single-cysteine mutants showed diffused pattern of protein localization, without any signs of aggregation. As shown in Figure 7A, the highest level of intermolecular cross-linking was observed for Leu14Cys and Phe17Cys mutants, a lower level for Phe15Cys and Gly16Cys mutants, and very little cross-linking for Asn18Cys substitution. These results indicate that: a) the first two transmembrane domains of CD9 alone can mediate its dimerization, and b) the ***g ***and ***c ***residues of TM1 (e.g. Leu14 and Phe17, Figure 3) are likely to be part of the intermolecular interface.

Similarly, single-cysteine substitutions were made for residues Gly77, Gly80 and Ala81 in TM2; in addition, proteins carrying a single wild-type cysteine, Cys9, Cys78 or Cys79, were tested. No protein aggregation was observed for any of these single-cysteine mutants. As shown in Figure 7B, the relatively low level of intermolecular cross-linking of wild-type CD9 TM(1+2)-GFP protein was enhanced dramatically in single-cysteine TM2 mutants Gly80Cys and Ala81Cys. The Gly77Cys mutant also had an elevated level of cross-linking. In contrast, any of the three native cysteines (9, 78 and 79) produced level of cross-linking not much greater than the wild-type TM(1+2) protein. Similar results were obtained with cysteine-reactive cross-linker BMB (data not shown). Likewise, comparable results were obtained with single-cysteine mutants of untagged, full-length CD9, using CuP (Figure 7C) as well as DTME cross-linker (data not shown).

These cross-linking results for TM1 and TM2 are consistent with our model that places residues Leu14, Phe17 and Gly80 on the same side of the TM1-TM2 pair (Figure 6, panel C). The strong cross-linking with Leu14Cys, Phe17Cys and Gly80Cys places the intermolecular interface toward the ***c ***and ***g ***phases of the TM1 helix, and the ***g ***phase of the TM2 helix, away from its ***e ***and ***f ***faces containing wild-type cysteines 78 and 79.

**Figure 8:**
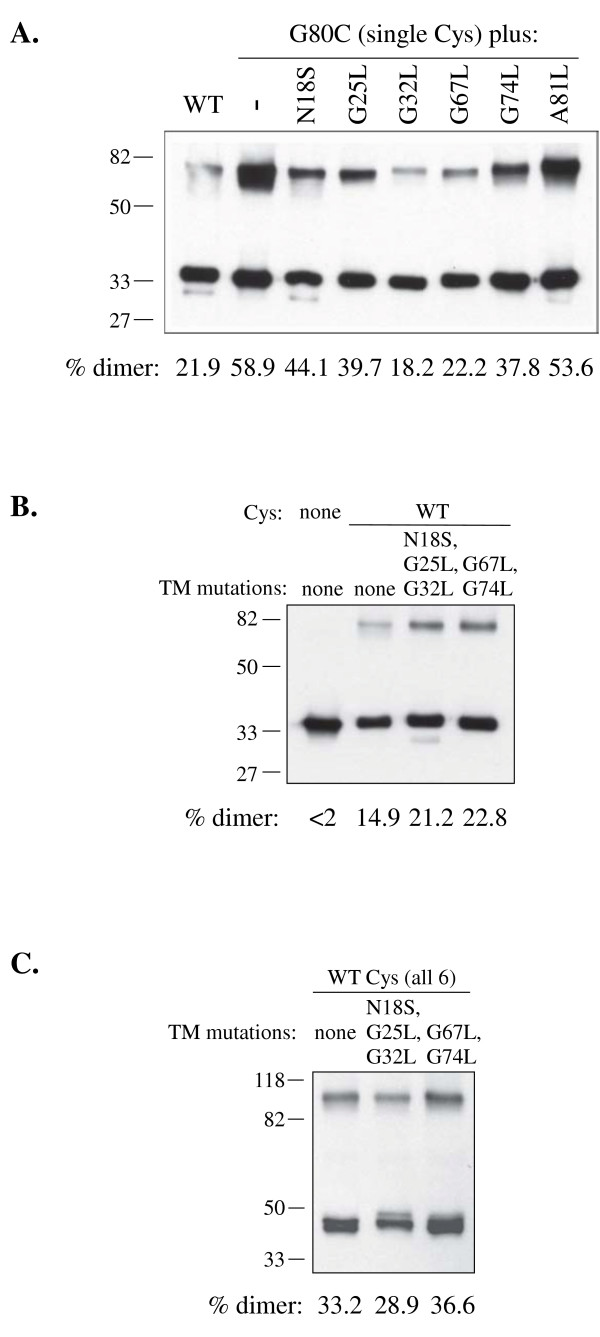
**Effect of mutations in conserved TM1 and TM2 residues on CD9 cross-linking**. RD cells were transfected with constructs encoding the following fusion proteins: panel **A**, CD9 TM(1+2)-GFP, either wild-type or single-cysteine G80C mutant, which also carries TM1 and TM2 substitutions indicated; panel **B**, CD9 TM(1+2)-GFP with no cysteines or wild-type cysteines (Cys9,78,79) plus TM1 and TM2 mutations; panel **C**, full-length CD9-GFP with TM1 and TM2 mutations indicated. The proteins were cross-linked and analyzed by GFP Western as in Figure 7. % dimer was calculated as for Figure 7.

Critical residues at the TM1-TM2 interface also affect dimerization indirectly. To assess specific CD9 dimerization, we used a Gly80Cys substitution at the intermolecular interface for cross-linking. As shown in Figure 8A, single replacements of conserved heptad residues in positions 18, 25, 32, 67 and 74 (Asn18Ser, Gly25/32/67/74→Leu) strongly decreased the cross-linking mediated by Cys80. The effect was most pronounced for mutations of residues, Gly32 and Gly67, located in the tightly packed extracellular end of TM helices (Figure 6). In contrast, mutations of residues closer to the cytoplasmic end of TM2 (Gly74 and especially Ala81) had only modest to very little effect on cross-linking.

Relatively low efficiency of intermolecular cross-linking via native residues Cys9, 78, and 79 (Figures 7B,C) correlates well with the predicted location of Cys78 and 79 away from the dimeric interface (Figure 3), and suggests that the extramembrane N-terminal part of CD9 (residues 1–13) does not self-associate. We next examined whether mutations of conserved Asn and Gly residues in TM1 and TM2 decreased low-level background cross-linking via native cysteines. As expected, these mutations had virtually no effect on dimer formation of CD9 TM(1+2)-GFP (Figure 8B). The level of covalent dimer formed was not diminished for triple Asn18Ser + Gly25Leu + Gly32Leu and double Gly67Leu + Gly74Leu mutants, compared to wild-type TM(1+2) CD9 molecule. Similarly, the same triple and double mutations in the context of full-length CD9-GFP protein (with six cysteines) produced wild-type levels of cross-linking (Figure 8C). We interpret these findings as evidence for at least two types of associations between CD9 molecules: primary, involving residues 14, 17 and 80, and dependent on integrity of conserved heptad residues in TM1 and TM2, and less efficient secondary interactions, probably representing random collision events, and independent of the heptad residues (see *Discussion *for more details).

### TM3 and TM4 cysteine residues in CD9 dimerization

**Figure 9:**
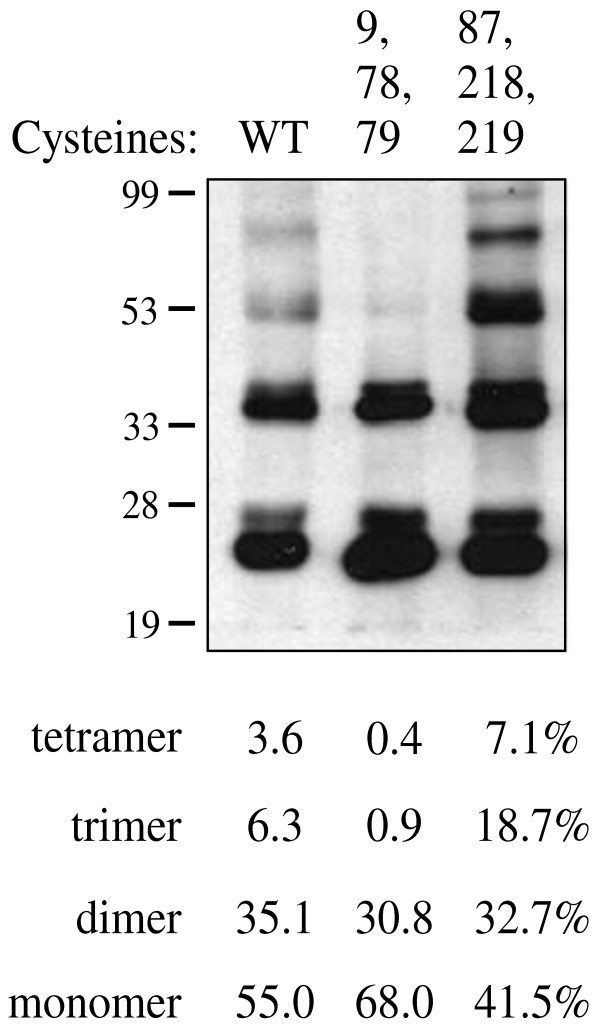
**CD9 cross-linking through N- or C-terminal cysteine residues**. RD cells were transfected with constructs encoding wild-type CD9 protein, CD9 containing only the three N-terminal cysteines (Cys9, 78 and 79), or the three C-terminal cysteines (Cys87, 218 and 219). Cells were treated with 2-BP for 16 hours and lysed in buffer containing 1% Brij97 for spontaneous disulfide cross-linking. Cell lysates were analyzed by CD9 Western. Relative intensity of bands corresponding to CD9 monomers, dimers, trimers and tetramers was calculated as a percent fraction of total protein material (monomers through tetramers).

After identifying the roles of conserved TM1 and TM2 residues in CD9 dimerization, we next probed whether residues proximal to TM domains 3 and 4 are also involved. To this end, disulfide cross-linking of full-length CD9 molecules containing 3 C-terminal cysteines (87, just before TM3; 218 and 219 in TM4) or 3 N-terminal cysteines (9, 78 and 79) was compared (Figure 9). We found that the C-terminal cysteines were only slightly better than N-terminal cysteines with respect to detection of CD9 dimers. However, markedly more trimers and tetramers were detected using C-terminal cysteines. Thus, residues 87, 218 and 219 at TM3 and TM4 in CD9 can together form contacts across the dimeric interface and also additional contacts with other neighboring CD9 molecules.

## Discussion

Here we provide the first detailed analysis of tetraspanin protein transmembrane domains. First, we show 1) the presence of a heptad repeat motif in TM1 and TM2, containing highly conserved Asn and Gly residues, 2) a leucine and glutamate/glutamine-containing heptad motif in TM3, and 3) high variability and absence of heptad repeats in TM4 sequences. Second, we provide evidence for a specific, intramolecular interaction between TM1 and TM2 domains, in which bulky hydrophobic residues pack against GG7 motif, and present a molecular model for this interaction. Third, experimental mapping of the CD9 dimerization interface firmly establishes an additional role for conserved TM1 and TM2 residues in dimeric intermolecular interactions. Fourth, preliminary evidence is provided to suggest that TM3 and TM4 domains contribute to expansion of CD9 dimers into higher order multimers.

### Conserved residues in TM1 and TM2 of tetraspanins: role in intramolecular packing

We hypothesized that the first two transmembrane domains of tetraspanins might interact with each other because: a) consecutive TM domains frequently associate in known protein 3D structures [35], and b) they both contain a series of highly conserved amino acids – several Gly residues and an Asn residue (Figure 1). Conserved Gly residues are a frequent theme in the organization of interacting transmembrane domains. Analysis of 3D helix packing in polytopic membrane proteins reveals that Gly residues tend to localize in buried positions, especially at the helix-helix interfaces and helix crossing points [37,38]. Due to the absence of a side chain, Gly provides a flat surface for packing of a side chain from another residue, without loss of side-chain entropy upon interaction. The most common Gly-containing motif is GxxxG [39,40]. In glycophorin A (GpA), the major glycoprotein in erythrocyte cell membranes, Gly79 and Gly83 are part of the LIxxGVxxGVxxT sequence that promotes homodimerization of parallel transmembrane α-helixes [41,42]. In the GpA dimerization motif, Gly residues allow for tight packing in the right-handed helical crossing [43]. There are also examples of left-handed helical crossing in the context of a GxxxG motif [44]. Other membrane proteins that use the GxxxG motif for homo- or heterodimerization include bacteriophage M13 coat proteins [45], yeast alpha factor receptor [46], integrin α IIb subunit [47], and ErbB1 receptor tyrosine kinase [48]. Other small residues, such as Ala and Ser, can substitute for Gly in this motif [49].

A protein motif in which Gly residues are separated by 6 other residues (GG7) is also common in transmembrane helices, especially in transporter/channel-like membrane proteins [50]. However, the structural features associated with this motif are not well known. In particular, it is unclear whether left-handed GG7 heptad repeat motif (as opposed to the "classic" right-handed GxxxG motif) can drive membrane helix association. In a recent work addressing this issue, Lear *et al. *[51] showed that a synthetic peptide containing Gly at heptad positions ***a ***and ***d ***could self-associate *in vitro*, likely in an antiparallel orientation. Heptad repeats containing conserved Gly residues occur in TM domains of α and β chains of MHC class II proteins, and mutations of the Gly residues disrupt the αβ heterodimer [52]. These examples demonstrate that Gly-based heptad motifs may be used for both intra- and intermolecular associations.

In this work, we identified a highly conserved GG7 motif in the first two tetraspanin TM domains. The GG7 sequence in tetraspanins is a part of a larger motif that also includes a conserved Asn residue in TM1 (NGG7) and an Ala/Ser/Thr residue in TM2 (GGA7). The seven-residue periodicity of these motifs strongly suggests their involvement in left-handed coiled coil packing reminiscent of the leucine zipper, rather than right-handed packing of the GpA-like GxxxG motif. For antiparallel helices, the left-handed crossing is in fact predominant over the right-handed in known TM domain structures [44].

In our model, heptad Gly residues in NGG7 and GGA7 sequences provide specific packing between antiparallel tetraspanin TM1 and TM2 helices by allowing tight van der Waals interactions with large hydrophobic residues (Figure 6). Highly efficient packing of bulky side chains against glycine residues is observed in known transmembrane protein 3D structures [38,53,54]. An example includes packing of helices M1 and M2 in potassium channel KcsA, where Val91 in M2 is paired with Gly43 in M1, and Leu36 in M1 contacts Ala98 and Gly99 in helix M2 [54,55]. In addition to facilitating helix-helix packing, Gly residues frequently provide additional C_α_H^...^O hydrogen bonds between two helices [44]. In our model, two C_α_-backbone carbonyl H-bonds are predicted – between residues Gly27-Gly67, and Trp22-Met70.

Although polar and charged amino acid residues (such as Asn in the TM1 heptad motif) are infrequent in transmembrane domains, they are functionally important. Polar residues such as glutamine, glutamic acid, aspartic acid and asparagine can promote strong oligomerization of model membrane-associated helices [56-58]. Ruan *et al. *[59] used asparagine scanning mutagenesis to probe the interface of self-associating polyleucine helices by detecting their enhanced self-interaction *in vitro *and in the *E. coli*-based ToxR assay. Thus, a hydrogen bond in an apolar environment can result in strong, though not necessarily specific, association of transmembrane helices. In fact, mutations to polar residues in transmembrane proteins are commonly associated with disease [60]. Because of this potential for non-specific interactions, polar residues tend to localize at buried positions in TM domains.

In our case, the conserved Asn18 residue in CD9 is predicted to be a part of the TM1-TM2 interface, though our model does not predict any electrostatic interaction between Asn18 and TM2 (Figure 6). Consistently, substitution such as Asn18Tyr (and Gly77Leu) in TM(1+2)-GFP protein was not destabilizing as analyzed by protein aggregation. Curiously, the full-length Asn18Ser CD9 migrated slightly slower on SDS-PAGE gel (data not shown), suggesting that Asn18 does play a role in maintaining conformation of the molecule. The Asn18Cys single-cysteine mutant shows very little intermolecular cross-linking (Figure 7A), supporting the proposed location of this residue at the intramolecular interface. It is tempting to speculate that the "pocket" between TM1 and TM2 lined by Asn18 and Gly77 might be important for accommodating palmitate moieties that target Cys78 and Cys79 residues, and/or important for access by the putative palmitoyl transferase to those residues. Understanding the exact role of these highly conserved Asn18 and Gly77 residues in tetraspanins awaits further investigation.

In summary, we identified conserved glycine residues of TM1 and TM2 of tetraspanins as key elements required for intramolecular packing. Mutations of these key residues (Gly25, Gly32, Gly67 and Gly74 in CD9) resulted in protein destabilization and aggregation. There is ample evidence in the literature for mutations in transmembrane proteins that lead to protein destabilization, misassembly and pathologic conditions [61]. Thus, we have identified conserved heptad Gly residues in TM1 and TM2 of tetraspanins as plausible targets of destabilizing mutations with potential functional consequences.

### Intermolecular interactions in tetraspanins

Tetraspanin CD9 forms mostly homodimers, but also a low level of heterodimers with CD81 and CD151 [25]. Thus, mapping the dimerization interface is an important next step in structure-function analysis of tetraspanins. Disulfide-mediated cross-linking, often in combination with cysteine-scanning mutagenesis, is a common strategy to probe oligomerization or intersubunit interactions of transmembrane proteins such as histidine kinase EnvZ [62], M(3) muscarinic acetylcholine receptor [63], *E. coli *lactose permease [64], synaptobrevin [65], integrins [66] and many others. In tetraspanins such as CD9, membrane-proximal cysteine residues are especially useful targets for disulfide trapping, as their linkage can be enhanced by pre-treating cells with 2-BP. While the ability of wild-type cysteines in CD9 to be cross-linked may indicate that they are close to the dimerization interface, more precise mapping was achieved here using cysteine-scanning mutagenesis.

Our data clearly identify regions, near the cytoplasmic face of TM1 and TM2, important for dimerization. Intermolecular zero-length cross-linking was highest when single cysteines were placed in positions 14, 17, 77, 80 and 81 in TM(1+2)-GFP molecule, or at positions 77, 80 or 81 in the full-length CD9 protein. Positions 14, 17 and 80 are distinct from the intramolecular interface and are on the same side of the TM1-TM2 pair (Figure 6). Thus, they are well-positioned to participate in an interaction with another molecule. At the same time, the model predicts that wild-type cysteines (Cys78, 79), which do not yield very efficient zero-length cross-linking, are on the other side of TM1-TM2 pair.

While using the cysteine at position 80 as the dimeric interface probe, mutations of conserved residues in TM1 and TM2 (especially Gly32 and Gly67 to Leu) clearly reduced intermolecular cross-linking. We do not suggest that those residues are directly involved in intermolecular interaction. Rather, we propose that destabilization of the intramolecular TM1-TM2 interaction by Gly to Leu substitutions (discussed above) causes an overall conformational change that reduces dimer formation.

An Ala81Leu mutation did not reduce cross-linking via Cys80, even though single-cysteine Ala81Cys molecules themselves produced a high level of cross-linking. These results, together with data on Gly32Leu and Gly67Leu mutations, are consistent with our model predicting that helices 1 and 2 interact more tightly near the extracellular end and less at the cytoplasmic end. This would give more flexibility to a cysteine at position 81 and also limit the effect of an Ala81Leu mutation. Location of this residue at the membrane/cytoplasmic border could also make it more accessible to CuP reagent as compared to residues buried in TM domain, thus elevating the efficiency of disulfide formation of the Ala81Cys mutant.

### Multiple interfaces in tetraspanin molecules

**Figure 10:**
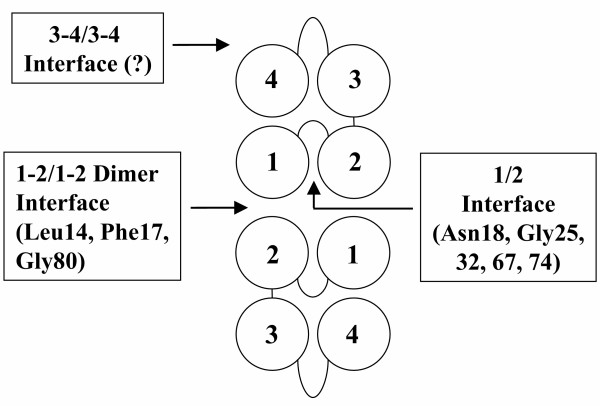
**A model of the organization of a tetraspanin dimer**. Two interfaces, the intramolecular TM1/TM2 and intermolecular TM1-TM2/TM1-TM2, have been analysed in this work. A third interface, intermolecular TM3-TM4/TM3-TM4, is predicted.

In the full-length CD9 molecules, the 3 C-terminal cysteines (Cys87, 218 and 219) located at or in TM3 and TM4 promoted efficient dimer and even more efficient oligomer formation compared to the 3 N-terminal cysteines (Figure 9). Cys87 alone can be used to capture CD9 dimers [25]. These results suggest the existence of two dimeric interfaces in CD9 molecule – the TM 1-2/1-2 interface and the TM 3-4/3-4 interface (Figure 10). In a TM(1+2) molecule, the destabilization of 1–2 interaction, e.g. by Gly→Leu mutations, would affect the 1-2/1-2 interface, as discussed above. However, these mutations would not interfere with the 3-4/3-4 interface in a full-length molecule, which includes Cys87, 218 and 219. Thus, cross-linking of full-length molecules, containing all 6 cysteine residues, would be unaffected, as seen in Figure 8C. Furthermore, wild-type Cys9, Cys78 and Cys79 are apparently not at the primary 1-2/1-2 interface. Their relative inefficiency in cross-linking CD9 TM(1+2) protein likely reflects weak secondary contacts between the molecules, or possibly random collision events. Such events should be independent of mutations in the conserved Gly residues in TM1 and TM2, as was demonstrated in Figure 8B. The potential existence of two interfaces in tetraspanin molecules, 1-2/1-2 and 3-4/3-4, should provide enhanced flexibility for forming additional intermolecular contacts. Current understanding of tetraspanin microdomains assumes a few strong, primary homotypic and heterotypic tetraspanin complexes (e.g. CD9-CD9, CD9-CD81, CD151-α3 integrin, CD81-EWI2) that help bring together various other proteins, forming secondary-type associations. Such properties of tetraspanins may bring signaling molecules such as protein kinase C or phosphatidylinositol 4-kinase to the vicinity of integrins [67,68].

The organization of the TM3 domain points to a potential role in protein-protein interactions. A motif of Leu-Leu-Glu(Gln) spaced 7 residues apart (heptad positions ***a***), with highly conserved residues in two consecutive positions ***d***, poses as a likely interaction module. If responsible for heterologous protein-protein interactions, it would form another distinct interface of tetraspanin molecule. Our preliminary data indicate that replacing the Leu and Glu residues in TM3 of CD9 with Ala has no effect on cell surface expression of the protein and its dimerization (data not shown). It remains to be tested if interactions with other proteins will be affected. Similarly, the TM4 domain may provide additional contributions to lateral tetraspanin associations. Much higher sequence variability, and the lack of a distinct heptad pattern suggests that TM4 is a major contributor to diversity among tetraspanin complexes. Structure-function analysis of TM3 and TM4 domains in tetraspanins is the subject of ongoing investigation.

## Conclusion

We have defined the TM1-TM2 intramolecular interface in tetraspanin CD9, providing evidence for glycines (Gly25 and Gly32 in TM1, Gly67 and Gly74 in TM2) packing against apposing bulky aliphatic residues. Second, we mapped an intermolecular CD9 interface (involved in CD9 homodimer formation) to the vicinity of residues Leu14 and Phe17 in TM1 and Gly77, Gly80 and Ala81 in TM2. Finally, we provide preliminary evidence that TM3 and TM4 in CD9 may contribute to a second intermolecular interface. Key CD9 residues involved in intra- and intermolecular interactions are highly conserved throughout the tetraspanin family, thus suggesting that our findings will apply to most tetraspanins.

## Methods

### Materials

Cell culture reagents were from Invitrogen (Carlsbad, CA). 2-bromopalmitate was from Fluka (Milwaukee, WI), N-ethylmaleimide (NEM) and 1,10-phenanthroline were from Sigma-Aldrich (St. Louis, MO), and chemical cross-linkers dithio-*bis*-maleimidoethane (DTME) and 1,4-*Bis*-maleimidobutane (BMB) were purchased from Pierce Endogen (Rockford, IL). Triton X-100, protease inhibitor cocktail and FuGENE 6 transfection reagent were obtained from Roche (Indianapolis, IN). Restriction endonucleases and *Pfu *DNA polymerase were obtained New England Biolabs (Beverly, MA) and Stratagene (Carlsbad, CA), respectively. All other chemicals were purchased from Sigma-Aldrich or Fisher Scientific (Pittsburg, PA).

### Sequence analysis

Tetraspanin sequences were obtained from SWISS-PROT and GenBank databases. Locus designations, accession numbers and the most commonly used protein names are summarized in Tables 1 and 2. TM segments were delineated by inspection of hydrophobicity profiles, using database annotations and previous analyses of TM sequences as a guide ([28], M. Hemler, unpublished), and aligned manually. Residue numbers in human CD9 sequence are used a reference point throughout the study.

### DNA cloning and mutagenesis

Sequence encoding CD9 protein was cloned into vector pcDNA3 (Invitrogen, Carlsbad, CA) and pEGFP-N1 (Clontech, Palo Alto, CA), for expression of untagged and C-terminally GFP-tagged CD9, respectively. pEGFP-N1 encoding CD9 TM(1+2) -GFP fusion protein was constructed by subcloning DNA for residues 1–83 of CD9 into *HindIII *and *PstI *sites of the vector; to introduce the *PstI *site, codon GTG for Val82 was changed to CTG (coding for Ala). In the resulting fusion protein, there is a 13-amino acid linker (with no cysteines) between CD9 and GFP. To minimize the low inherent ability of GFP to homodimerize, which could potentially influence the results of CD9 cross-linking, we used a monomeric GFP mutant, Leu221→Lys [34], for cross-linking experiments.

Mutations were introduced in full-length and TM(1+2) CD9 proteins by a PCR-based strategy using mutagenic primers and *Pfu *DNA polymerase. All mutations were confirmed by DNA sequencing.

### Protein expression, microscopy, cysteine disulfide cross-linking and Western blotting

DNA constructs encoding TM(1+2)-GFP or full-length CD9 proteins were transfected into human rhabdomyosarcoma RD cells using the FuGENE 6 reagent. Cells expressing GFP fusion proteins were analysed by fluorescence microscopy 18–28 hours post-transfection. Images were captured using Spot 1.4.0 camera (Diagnostic Instruments, Sterling Heights, MI) attached to Nikon Eclipse TE300 microscope.

For experiments involving cysteine-mediated cross-linking, cells were treated with 50 μM 2-BP starting 24–26 hours post-transfection and continuing for 16–18 hours. Cross-linking was carried out by incubating cells in HBSM buffer (25 mM Hepes-NaOH, pH 7.2, 150 mM NaCl, 2 mM MgCl_2_) containing either a) 0.6 mM CuSO_4 _and 1.8 mM 1,10-phenanthroline (CuP complex) or b) 0.2 mg/ml homobifunctional cysteine-reactive cross-linker (e.g. DTME), diluted from fresh 10 mg/ml solution in DMSO. After incubation for 10–15 minutes (with CuP) or 30–45 minutes (with cross-linker), cells were washed twice for 10 minutes with HBSM containing 10 mM NEM to block residual free cysteines. Cells were lysed in HBSM containing 1% Triton X-100, 0.1% SDS and a cocktail of protease inhibitors with 1 mM EDTA at 4°C for 45–60 minutes. Cell lysate was clarified by centrifugation at 14,000 × g for 15 minutes, an aliquot was removed, and proteins from it were precipitated by addition of trichloroacetic acid to 10% on ice followed by centrifugation at 14,000 × g for 10 minutes. After two washes with ice-cold acetone, protein pellet was solubilized in SDS-PAGE sample buffer without a reducing agent (50 mM Tris-HCl, pH 6.8, 1% SDS, 8% glycerol).

In some experiments, CD9 protein was immunoprecipitated using monoclonal antibody Alb6 (Immunotech, Marseille, France). Proteins were separated by SDS-PAGE and analyzed by Western blotting using monoclonal antibody JL-8 (Clontech) for GFP or Alb6 for CD9. Bands from X-ray films were quantitated using GeneTools™ software from Syngene (Frederick, MD).

### Modeling of TM1-TM2 interaction

An atomic model of the CD9 TM1-TM2 dimer was constructed with a Monte Carlo-simulated annealing (MCSA) algorithm [69]. Two idealized α-helices corresponding to TM1 residues Tyr12 through Leu35 and TM2 residues Gly59 through Val82 were docked with six orthogonal parameters: three rigid body translations and three rotations. During each step of a MCSA cycle, there was an equal probability of changing either one parameter or all six parameters to random values. A conformation's energy was calculated *in vacuo *with the AMBER united-atom force field for van der Waals interactions [70]. The van der Waals term was modified as described by Kuhlman and Baker [71]. If a structure had favorable dimerization energy, the energies of select mutants were calculated. Structures were selected with a novel scoring function that maximizes the Boltzmann probability of dimerization for silent mutations while minimizing the probability for disruptive mutations. Asn18Ser, Gly77Leu, and Gly80Leu were scored as silent mutations while Gly25Leu, Gly32Leu, Gly67Leu, and Gly74Leu were considered to be disruptive. Each MCSA cycle consisted of 50,000 steps with an exponential temperature decay from 10,000 to 10 K.

Ten MCSA cycles through global sample space were used to restrict the search area. Parameters were restricted to ± 2 standard deviations from their mean value for structures within 10 kcal of the best structure. MCSA cycles were repeated as described above with additional optimization of χ values: rotamers at the protein-protein interface were optimized with Dead End Elimination [72], and χ values were further optimized with Monte Carlo. All MCSA cycles converged upon structures that were within a root mean squared deviation (RMSD) of 1.5 Å with the best structure, and structures that scored within 5 kcal of the best score had an RMSD of less than 0.5 Å with the best structure.

## List of abbreviations

2-BP, 2-bromopalmitate; CuP, Cu^2+^-phenanthroline; DTME, dithio-bis-maleimidoethane; LEL, large extracellular loop; NEM, N-ethylmaleimide; TM, transmembrane (domain).

## Authors' contributions

OVK carried out sequence comparisons, mutational analysis and cross-linking experiments, and drafted the manuscript. DGM built the TM1-TM2 interaction model and contributed to the manuscript. WFD supervised DGM's work. MEH coordinated the whole study and prepared the final manuscript.

## Figures and Tables

**Table 1:** Human tetraspanin sequences analyzed in this study.

SWISS-PROT locus name	Accession number	Protein name(s)
TSN1_HUMAN	O60635	NET-1, Tspan-1
CD82_HUMAN	P27701	CD82 antigen NM_030927
CD37_HUMAN	P11049	CD37 antigen
CD53_HUMAN	P19397	CD53 antigen
T4S7_HUMAN	O14817	NAG-2, Tspan-4
TNE5_HUMAN	O75954	NET-5
BAB55318	AK027715	-
TSN2_HUMAN	O60636	Tspan-2
T4S9_HUMAN	P62079	NET-4, Tspan-5
TM4B_HUMAN	Q9UKR8	TM4B
-	NM_030927	-
C151_HUMAN	P48509	CD151 antigen, PETA-3
CD9_HUMAN	P21926	CD9 antigen, p24, MRP-1
CD81_HUMAN	P60033	CD81 antigen, TAPA-1
T4S3_HUMAN	P19075	CO-029 antigen
T4S8_HUMAN	O60637	Tspan-3, TM4-A
T412_HUMAN	O95859	NET-2
SAS_HUMAN	Q12999	Sarcoma amplified sequence (SAS)
T413_HUMAN	O95857	NET-6
TNE7_HUMAN	O95858	NET-7
CD63_HUMAN	P08962	CD63 antigen, LAMP-3
T4S2_HUMAN	P41732	A15, TALLA-1, CD231 antigen
T4S6_HUMAN	O43657	Tspan-6, TM4-D
OCSP_HUMAN	Q9H1Z9	Oculospanin (Ocsp)
UPKA_HUMAN	O00322	Uroplakin Ia (UPIa)
UPKB_HUMAN	O75841	Uroplakin Ib (UPIb)
RDS_HUMAN	P23942	Peripherin, Retinal degeneration slow protein (RDS)
ROM1_HUMAN	Q03395	Rod outer segment membrane protein-1 (ROM-1)

**Table 2:** Vertebrate CD9 orthologs analyzed in this study.

Organism	SWISS-PROT or GenBank locus name	Accession number
*Homo sapiens *(Human)	CD9_HUMAN	P21926
*Cercopithecus aethiops *(African green monkey)	CD9_CERAE	P30409
*Sus scrofa *(Pig)	CD9_PIG	Q8WMQ3
*Bos taurus *(Cow)	CD9_BOVIN	P30932
*Felis catus *(Cat)	CD9_FELCA	P40239
*Rattus norvegicus *(Norway rat)	CD9_RAT	P40241
*Mus musculus *(House mouse)	CD9_MOUSE	P40240
*Gallus gallus *(Chicken)	NP_990093 (GenBank)	NP_990093
*Petromyzon marinus *(Sea lamprey)	AAN64299 (GenBank)	AAN64299
*Danio rerio *(Zebrafish)	AAH59691 (GenBank)	AAH59691

## References

[B1] Stipp CS, Kolesnikova TV, Hemler ME (2003). Functional domains in tetraspanin proteins. Trends Biochem Sci.

[B2] Hemler ME (2003). Tetraspanin proteins mediate cellular penetration, invasion and fusion events, and define a novel type of membrane microdomain. Ann Rev Cell Dev Biol.

[B3] Berditchevski F (2001). Complexes of tetraspanins with integrins: more than meets the eye. J Cell Sci.

[B4] Tarrant JM, Robb L, van Spriel AB, Wright MD (2003). Tetraspanins: molecular organisers of the leukocyte surface. Trends Immunol.

[B5] Le Naour F, Rubinstein E, Jasmin C, Prenant M, Boucheix C (2000). Severely reduced female fertility in CD9-deficient mice. Science.

[B6] Kaji K, Oda S, Shikano T, Ohnuki T, Uematsu Y, Sakagami J, Tada N, Miyazaki S, Kudo A (2000). The gamete fusion process is defective in eggs of CD9-deficient mice. Nature Genet.

[B7] Miyado K, Yamada G, Yamada S, Hasuma H, Nakamura Y, Ryu F, Suzuki K, Kosai K, Inoue K, Ogura A, Okabe M, Mekada E (2000). Requirement of CD9 on the egg plasma membrane for fertilization. Science.

[B8] Maecker HT, Levy S (1997). Normal lymphocyte development but delayed humoral immune response in CD81-null mice. J Exp Med.

[B9] Miyazaki T, Muller U, Campbell KS (1997). Normal development but differentially altered proliferative responses of lymphocytes in mice lacking CD81. EMBO J.

[B10] Wright MD, Geary SM, Fitter S, Moseley GW, Lau LM, Sheng KC, Apostolopoulos V, Stanley EG, Jackson DE, Ashman LK (2004). Characterization of mice lacking the tetraspanin superfamily member CD151. Mol Cell Biol.

[B11] Kazarov AR, Yang X, Stipp CS, Sehgal B, Hemler ME (2002). An extracellular site on tetraspanin CD151 determines α 3 and α 6 integrin-dependent cellular morphology. J Cell Biol.

[B12] Zhu GZ, Miller BJ, Boucheix C, Rubinstein E, Liu CC, Hynes RO, Myles DG, Primakoff P (2002). Residues SFQ (173–175) in the large extracellular loop of CD9 are required for gamete fusion. Development.

[B13] Kitadokoro K, Bordo D, Galli G, Petracca R, Falugi F, Abrignani S, Grandi G, Bolognesi M (2001). CD81 extracellular domain 3D structure: insight into the tetraspanin superfamily structural motifs. EMBO J.

[B14] Seigneuret M, Delaguillaumie A, Lagaudriere-Gesbert C, Conjeaud H (2001). Structure of the tetraspanin main extracellular domain. A partially conserved fold with a structurally variable domain insertion. J Biol Chem.

[B15] Maecker HT, Todd SC, Levy S (1997). The tetraspanin superfamily: molecular facilitators. FASEB J.

[B16] Rubinstein E, Le Naour F, Lagaudrière-Gesbert C, Billard M, Conjeaud H, Boucheix C (1996). CD9, CD63, CD81, and CD82 are components of a surface tetraspan network connected to HLA-DR and VLA antigens. Eur J Immunol.

[B17] Claas C, Stipp CS, Hemler ME (2001). Evaluation of prototype TM4SF protein complexes and their relation to lipid rafts. J Biol Chem.

[B18] Charrin S, Manie S, Billard M, Ashman L, Gerlier D, Boucheix C, Rubinstein E (2003). Multiple levels of interactions within the tetraspanin web. Biochem Biophys Res Commun.

[B19] Berditchevski F, Odintsova E, Sawada S, Gilbert E (2002). Expression of the palmitoylation-deficient CD151 weakens the association of alpha 3beta 1 integrin with the tetraspanin-enriched microdomains and affects integrin-dependent signalling. J Biol Chem.

[B20] Charrin S, Manie S, Oualid M, Billard M, Boucheix C, Rubinstein E (2002). Differential stability of tetraspanin/tetraspanin interactions: role of palmitoylation. FEBS Lett.

[B21] Yang X, Claas C, Kraeft SK, Chen LB, Wang Z, Kreidberg JA, Hemler ME (2002). Palmitoylation of tetraspanin proteins: modulation of CD151 lateral interactions, subcellular distribution, and integrin-dependent cell morphology. Mol Biol Cell.

[B22] Berditchevski F, Gilbert E, Griffiths MR, Fitter S, Ashman L, Jenner SJ (2001). Analysis of the CD151-alpha3beta1 integrin and CD151-tetraspanin interactions by mutagenesis. J Biol Chem.

[B23] Cannon KS, Cresswell P (2001). Quality control of transmembrane domain assembly in the tetraspanin CD82. EMBO J.

[B24] Toyo-Oka K, Yashiro-Ohtani Y, Park CS, Tai XG, Miyake K, Hamaoka T, Fujiwara H (1999). Association of a tetraspanin CD9 with CD5 on the T cell surface: role of particular transmembrane domains in the association. Int Immunol.

[B25] Kovalenko OV, Yang X, Kolesnikova TV, Hemler ME (2004). Evidence for specific tetraspanin homodimers: inhibition of palmitoylation makes cysteine residues available for cross-linking. Biochem J.

[B26] Boucheix C, Thien Duc GH, Jasmin C, Rubinstein E (2001). Tetraspanins and malignancy. Exp Rev Mol Med.

[B27] Hemler ME (2001). Specific tetraspanin functions. J Cell Biol.

[B28] Bienstock RJ, Barrett JC (2001). KAI1, a prostate metastasis suppressor: prediction of solvated structure and interactions with binding partners; integrins, cadherins, and cell-surface receptor proteins. Mol Carcinog.

[B29] Burkhard P, Stetefeld J, Strelkov SV (2001). Coiled coils: a highly versatile protein folding motif. Trends Cell Biol.

[B30] Langosch D, Heringa J (1998). Interaction of transmembrane helices by a knobs-into-holes packing characteristic of soluble coiled coils. Proteins.

[B31] Lupas A (1996). Coiled coils: new structures and new functions. Trends Biochem Sci.

[B32] Walshaw J, Woolfson DN (2003). Extended knobs-into-holes packing in classical and complex coiled-coil assemblies. J Struct Biol.

[B33] O'Shea EK, Klemm JD, Kim PS, Alber T (1991). X-ray structure of the GCN4 leucine zipper, a two-stranded, parallel coiled coil. Science.

[B34] Zacharias DA, Violin JD, Newton AC, Tsien RY (2002). Partitioning of lipid-modified monomeric GFPs into membrane microdomains of live cells. Science.

[B35] Bowie JU (1997). Helix packing in membrane proteins. J Mol Biol.

[B36] Snapp EL, Hegde RS, Francolini M, Lombardo F, Colombo S, Pedrazzini E, Borgese N, Lippincott-Schwartz J (2003). Formation of stacked ER cisternae by low affinity protein interactions. J Cell Biol.

[B37] Eilers M, Shekar SC, Shieh T, Smith SO, Fleming PJ (2000). Internal packing of helical membrane proteins. Proc Natl Acad Sci USA.

[B38] Javadpour MM, Eilers M, Groesbeek M, Smith SO (1999). Helix packing in polytopic membrane proteins: role of glycine in transmembrane helix association. Biophys J.

[B39] Russ WP, Engelman DM (2000). The GxxxG motif: a framework for transmembrane helix-helix association. J Mol Biol.

[B40] Senes A, Gerstein M, Engelman DM (2000). Statistical analysis of amino acid patterns in transmembrane helices: the GxxxG motif occurs frequently and in association with beta-branched residues at neighboring positions. J Mol Biol.

[B41] Lemmon MA, Flanagan JM, Hunt JF, Adair BD, Bormann BJ, Dempsey CE, Engelman DM (1992). Glycophorin A dimerization is driven by specific interactions between transmembrane alpha-helices. J Biol Chem.

[B42] Lemmon MA, Treutlein HR, Adams PD, Brunger AT, Engelman DM (1994). A dimerization motif for transmembrane alpha-helices. Nature Struct Biol.

[B43] MacKenzie KR, Prestegard JH, Engelman DM (1997). A transmembrane helix dimer: structure and implications. Science.

[B44] Senes A, Ubarretxena-Belandia I, Engelman DM (2001). The Calpha – H^...^O hydrogen bond: a determinant of stability and specificity in transmembrane helix interactions. Proc Natl Acad Sci USA.

[B45] Deber CM, Khan AR, Li Z, Joensson C, Glibowicka M, Wang J (1993). Val→Ala mutations selectively alter helix-helix packing in the transmembrane segment of phage M13 coat protein. Proc Natl Acad Sci USA.

[B46] Overton MC, Chinault SL, Blumer KJ (2003). Oligomerization, biogenesis, and signaling is promoted by a glycophorin A-like dimerization motif in transmembrane domain 1 of a yeast G protein-coupled receptor. J Biol Chem.

[B47] Li R, Gorelik R, Nanda V, Law PB, Lear JD, DeGrado WF, Bennett JS (2004). Dimerization of the transmembrane domain of integrin alphaIIb subunit in cell membranes. J Biol Chem.

[B48] Gerber D, Sal-Man N, Shai Y (2004). Two motifs within a transmembrane domain, one for homodimerization and the other for heterodimerization. J Biol Chem.

[B49] Kleiger G, Grothe R, Mallick P, Eisenberg D (2002). GXXXG and AXXXA: common alpha-helical interaction motifs in proteins, particularly in extremophiles. Biochemistry.

[B50] Liu Y, Engelman DM, Gerstein M (2002). Genomic analysis of membrane protein families: abundance and conserved motifs. Genome Biol.

[B51] Lear JD, Stouffer A, Gratkowski H, Nanda V, DeGrado WF (2004). Association of a model transmembrane peptide containing Gly in a heptad sequence motif. Biophys J.

[B52] Cosson P, Bonifacino JS (1992). Role of transmembrane domain interactions in the assembly of class II MHC molecules. Science.

[B53] Adamian L, Liang J (2001). Helix-helix packing and interfacial pairwise interactions of residues in membrane proteins. J Mol Biol.

[B54] Lee AG (2002). Ca^2+^-ATPase structure in the E1 and E2 conformations: mechanism, helix-helix and helix-lipid interactions. Biochim Biophys Acta.

[B55] Doyle DA, Morais CJ, Pfuetzner RA, Kuo A, Gulbis JM, Cohen SL, Chait BT, MacKinnon R (1998). The structure of the potassium channel: molecular basis of K+ conduction and selectivity. Science.

[B56] Zhou FX, Cocco MJ, Russ WP, Brunger AT, Engelman DM (2000). Interhelical hydrogen bonding drives strong interactions in membrane proteins. Nature Struct Biol.

[B57] Choma C, Gratkowski H, Lear JD, DeGrado WF (2000). Asparagine-mediated self-association of a model transmembrane helix. Nature Struct Biol.

[B58] Gratkowski H, Lear JD, DeGrado WF (2001). Polar side chains drive the association of model transmembrane peptides. Proc Natl Acad Sci USA.

[B59] Ruan W, Lindner E, Langosch D (2004). The interface of a membrane-spanning leucine zipper mapped by asparagine-scanning mutagenesis. Protein Sci.

[B60] Partridge AW, Therien AG, Deber CM (2002). Polar mutations in membrane proteins as a biophysical basis for disease. Biopolymers.

[B61] Sanders CR, Myers JK (2004). Disease-related misassembly of membrane proteins. Annu Rev Biophys Biomol Struct.

[B62] Cai SJ, Khorchid A, Ikura M, Inouye M (2003). Probing catalytically essential domain orientation in histidine kinase EnvZ by targeted disulfide crosslinking. J Mol Biol.

[B63] Hamdan FF, Ward SD, Siddiqui NA, Bloodworth LM, Wess J (2002). Use of an in situ disulfide cross-linking strategy to map proximities between amino acid residues in transmembrane domains I and VII of the M3 muscarinic acetylcholine receptor. Biochemistry.

[B64] Guan L, Murphy FD, Kaback HR (2002). Surface-exposed positions in the transmembrane helices of the lactose permease of *Escherichia coli *determined by intermolecular thiol cross-linking. Proc Natl Acad Sci USA.

[B65] Roy R, Laage R, Langosch D (2004). Synaptobrevin transmembrane domain dimerization-revisited. Biochemistry.

[B66] Luo B-H, Springer TA, Takagi J (2004). A specific interface between integrin transmembrane helices and affinity for ligand. PLoS Biol.

[B67] Yauch RL, Hemler ME (2000). Specific interactions among transmembrane 4 superfamily (TM4SF) proteins and phosphatidylinositol 4-kinase. Biochem J.

[B68] Zhang XA, Bontrager AL, Hemler ME (2001). TM4SF proteins associate with activated PKC and link PKC to specific beta1 integrins. J Biol Chem.

[B69] Li W, Metcalf D, Gorelik R, Li R, Mitra N, Nanda V, Law PB, Lear JD, DeGrado WF, Bennett JS (2005). A push-pull mechanism for regulating integrin function. Proc Natl Acad Sci USA.

[B70] Weiner SJ, Kollman PA, Case DA, Singh UC, Ghio C, Alagona G, Profeta S, Weiner P (1984). A new force field for molecular mechanical simulation of nucleic acids and proteins. J Am Chem Soc.

[B71] Kuhlman B, Baker D (2000). Native protein sequences are close to optimal for their structures. Proc Natl Acad Sci USA.

[B72] Desmet J, De Maeyer M, Hazes B, Lasters I (1992). The dead-end elimination theorem and its use in protein side-chain positioning. Nature.

